# Astragaloside IV Attenuates Myocardial Ischemia-Reperfusion Injury from Oxidative Stress by Regulating Succinate, Lysophospholipid Metabolism, and ROS Scavenging System

**DOI:** 10.1155/2019/9137654

**Published:** 2019-06-24

**Authors:** Miaomiao Jiang, Jingyu Ni, Yuanlin Cao, Xiaoxue Xing, Qian Wu, Guanwei Fan

**Affiliations:** ^1^Tianjin State Key Laboratory of Modern Chinese Medicine, Tianjin University of Traditional Chinese Medicine, Tianjin, China; ^2^Tianjin Key Laboratory of Translational Research of TCM Prescription and Syndrome, Tianjin, China; ^3^Shanghai Center for Bioinformation Technology, Shanghai 201203, China; ^4^First Teaching Hospital of Tianjin University of Traditional Chinese Medicine, Tianjin Key Laboratory of Translational Research of TCM Prescription and Syndrome, Tianjin, China

## Abstract

Astragaloside IV is one of the main active ingredients isolated from *Astragalus membranaceus*. Here we confirmed its protective effect against cardiac ischemia-reperfusion (I/R) injury and aimed to investigate the potential molecular mechanisms involved. Pretreatment of *ex vivo* and *in vivo* I/R-induced rat models by astragaloside IV significantly prevented the ratio of myocardium infarct size, systolic and diastolic dysfunction, and the production of creatine kinase and lactate dehydrogenase. Metabolic analyses showed that I/R injury caused a notable reduction of succinate and elevation of lysophospholipids, indicating excessive reactive oxygen species (ROS) generation driven by succinate's rapid reoxidization and glycerophospholipid degradation. Molecular validation mechanistically revealed that astragaloside IV stimulated nuclear factor (erythroid-derived 2)-like 2 (Nrf2) released from Kelch-like ECH-associated protein 1 (Keap1) and translocated to the nucleus to combine with musculoaponeurotic fibrosarcoma (Maf) to initiate the transcription of antioxidative gene heme oxygenase-1 (HO-1), which performed a wide range of ROS scavenging processes against pathological oxidative stress in the hearts. As expected, increasing succinate and decreasing lysophospholipid levels were observed in the astragaloside IV-pretreated group compared with the I/R model group. These results suggested that astragaloside IV ameliorated myocardial I/R injury by modulating succinate and lysophospholipid metabolism and scavenging ROS via the Nrf2 signal pathway.

## 1. Introduction

In recent years, ischemic cardiomyopathy has been a public health concern with a rising incidence that results in high morbidity and mortality worldwide [1]. Acute myocardial infarction (AMI) has the most severe effect, and the aftereffect of cardiac disease happens in the wake of interruption on the blood supply to the myocardium. Timely reperfusion for ST-elevation myocardial infarction is the cornerstone of treatment to reduce the mortality for patients with AMI in the last decades [2]. Inevitably, reoxygenation of hypoxic tissue exacerbates injury experienced during the ischemic episode, which is further referred to as myocardial ischemia-reperfusion (I/R) injury [3, 4]. In clinical practice, I/R injury influences the therapeutic effect of the reperfusion strategy delivered to patients and has seriously adverse consequences, so that adjunctive medications aimed at reducing I/R injury are urgently needed.

Oxidative stress is one of the main pathogenic mechanisms of myocardial I/R injury [5]. Under normal conditions, a small amount of free radicals produced by oxidative metabolism in the organism can be removed in time by its own antioxidant system. During reperfusion of the ischemic myocardium, oxygen free radicals occur explosively and then initiate oxidative damage through their accumulation in the heart, which further results in destruction of oxidation balance and antioxidant systems, cell death, and aberrant immune responses [6, 7]. Study evidence has demonstrated that metabolism provides substrates for reactive oxygen species (ROS), and thus, ROS generation is significantly influenced by these metabolic substrates, such as glucose, lipids, succinate, acetoacetate, choline, and dimethylglycine [8–11]. Metabolic dysfunction in tissues and organs caused by I/R injury has been manifested via the changes of metabolic substances in the body fluid or tissue [12], indicating that modulating metabolic substrate utilization in the myocardium would be an interesting therapeutic strategy for attenuation of ROS-related biochemical and functional damage.

Astragaloside IV is one of the main active ingredients in *Astragalus membranaceus* with various activities such as antitumor [13], anti-inflammation [14], antioxidation [15], antidiabetes [16], anticerebral ischemia [17], and anticardiovascular diseases [18]. Potential mechanisms of its protective effect on the heart mainly involve regulating the expression of ATP-sensitive potassium channel subunits [19] and levels of ATP, ADP, and AMP in the myocardium [20], stimulating angiogenesis and accumulation of nitric oxide (NO) through Janus kinase 2 (JAK2)/signal transducer and activator of transcription 3 (STAT3) and the extracellular signal-regulated kinase 1/2 (ERK1/2) signaling pathway [21], upregulating the hypoxia-inducible factor-1*α* (HIF-1*α*) expression [22] and preventing mitochondrial permeability transition pores from opening up by inactivating the glycogen synthase enzyme-3*β* (GSK-3*β*) [23]. Whether astragaloside IV mediate the metabolic response to myocardial I/R injury and the specific molecular mechanisms responsible for scavenging effect to excessive ROS are not completely understood.

In this study, we have demonstrated that astragaloside IV alleviates cardiac damage induced by I/R using *ex vivo* and *in vivo* rat models. We further systematically investigate the effects of astragaloside IV intake on the metabolism of the rat myocardium using ^1^H nuclear magnetic resonance (^1^H NMR) spectroscopy in conjunction with ultra-high-performance liquid chromatography with quadrupole-orbitrap mass spectrometry (UHPLC-MS). A global metabolic profiling approach is used to screen for potential metabolites that may be associated with I/R-induced oxidative stress, and the alternations of these metabolites are employed to evaluate the cardioprotective effect of astragaloside IV. Furthermore, we also assess whether astragaloside IV intervention can eliminate excessive ROS by regulating oxidative stress-related proteins. Taken all together, this study provides extensive information of astragaloside IV against myocardial I/R injury that reveals the details of metabolism pathways and the enzymology involved.

## 2. Materials and Methods

### 2.1. Animals

Male Sprague-Dawley (SD) rats (SPF level, weighting 320 ± 20 g) were purchased from Beijing HFK Bioscience Co. Ltd., China (license No. SCXk2014-0004). All procedures were approved by the Animal Care and Use Committee of Tianjin University of Traditional Chinese Medicine and were in accordance with the Guide for the Care and Use of Laboratory Animals. The rats were housed in cages at a temperature of 22 ± 2°C and humidity of 40 ± 5% with standard diet and water available ad libitum on a 12-hour light/dark cycle. The animals were fasted for 12 hours before the experiment but allowed free access to water.

### 2.2. Chemicals

Astragaloside IV (purity ≥99%) was obtained from Shanghai Winherb Medical Technology Co. Ltd. (Shanghai, China). Deuterium oxide (D_2_O, 99.9% atom %D) and 3-(trimethylsilyl)-propionic-2,2,3,3-*d*_4_ acid sodium salt (TSP-*d*_4_, 98% atom %D) were purchased from Sigma-Aldrich (St. Louis, MO, USA). Lysophosphatidylcholine (12 : 0) and phosphatidylcholine (11 : 0/11 : 0) were purchased from Avanti Polar Lipids (Birmingham, AL, USA).

### 2.3. Langendorff Heart Experiments

**Figure 1:**
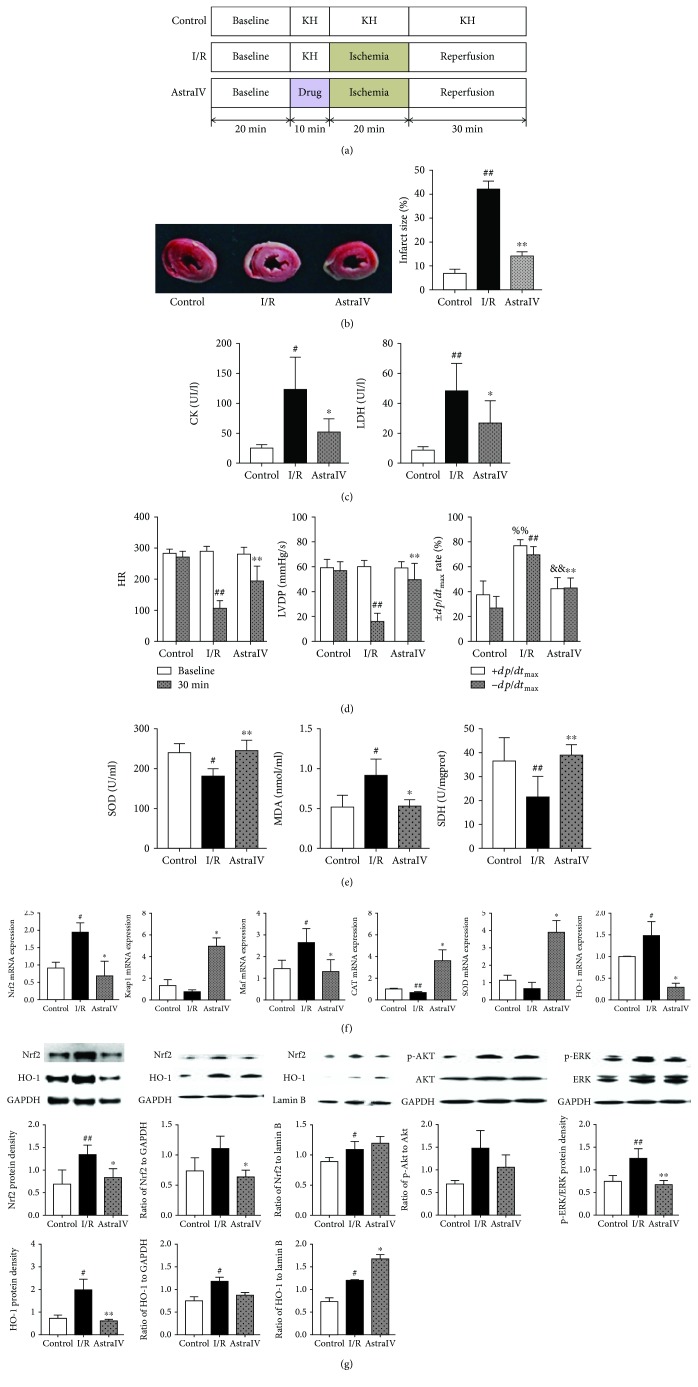


A total of 60 rats were anesthetized with 5% chloral hydrate (300 mg/kg i.p.). Thoracotomy was performed, and hearts were rapidly excised into an ice-cold Krebs-Henseleit (KH) buffer (118 mM NaCl, 24.0 mM NaHCO_3_, 4.7 mM KCl, 1.2 mM MgSO_4_, 1.2 mM KH_2_PO_4_, 11.1 mM glucose, and 2.5 mM CaCl_2_) at pH 7.4. After removal of the lungs and surrounding tissues, the aorta was attached to a perfusion apparatus where hearts were perfused at a constant pressure of 65 mmHg with 37°C KH buffer in carbogen (95% O_2_ and 5% CO_2_). After 20 min recovery of function and stabilization of rhythm, the hearts were separated into four groups: 60 min normoxic perfusion (control group), 10 min normoxic perfusion followed by 20 min global ischemia plus 30 min reperfusion (I/R group), and 10 min perfusion with 0.05 *μ*M astragaloside IV in KH buffer followed by 20 min global ischemia plus 30 min reperfusion (AstraIV group). The experimental design is shown in Figure 1(a). At the end of the experiment, hearts were snap-frozen in liquid nitrogen and sorted at -80°C.

The infracted areas were displayed as the area unstained by 2,3,5-triphenyltetrazolium chloride (TTC) and quantified by ImageJ software. Cardiac function parameters were monitored before ischemia and each time period of reperfusion (5, 10, 20, and 30 min), including heart rate (HR), coronary flow, left ventricular development pressure (LVDP), left ventricular end diastolic pressure, rate pressure product (RPP), left ventricular maximum upstroke velocity (+d*p*/d*t*_max_), and left ventricular maximum descent velocity (−d*p*/d*t*_max_). These parameters were recorded continuously on a computer using a PowerLab data acquisition system (8SP Chart 7 software; ADInstruments, Castle Hill, Australia). Levels of creatine kinase (CK), lactate dehydrogenase (LDH), superoxide dismutase (SOD), malondialdehyde (MDA), and succinate dehydrogenase (SDH) were determined by commercial kits according to the manufacturer's instructions on automatic biochemical detector (Microlab 300, Holland Rittal Science Co. Ltd., Holland) and microplate reader (EnSpire, PerkinElmer Co. Ltd., USA).

### 2.4. Metabolic Profiling Based on NMR Measurements

Frozen heart samples were ground into powder on dry ice, 50 mg of which was extracted with cold methanol and water (2 : 1, *v*/*v*) for 3 times. Combined extracts were dried in nitrogen and redissolved in 200 *μ*l D_2_O containing 1 mM TSP-*d*_4_. After centrifugation, 150 *μ*l supernatant was transferred into 3 mm NMR tube for testing. All NMR spectra were recorded at 298 K on a Bruker AVIII HD 600 MHz spectrometer (Bruker Company, Switzerland) operating at 600.25 MHz for proton resonance frequency. One-dimensional ^1^H NMR spectra of heart extracts were acquired with CPMGPR1D pulse sequences (from the Bruker pulse sequence library) with water signal presaturation. The time of relaxation delay was set to 4 s, and the 90° pulse length was adjusted to about 11.65 *μ*s. Sixty-four transients were recorded into 32k data points with spectral width of 12019.2 Hz.

Free induction decays (FID) were transformed using MestReNova 6.1.0 (Mestrelab Research S.L., Spain) and then phase- and baseline-corrected manually. The ^1^H NMR spectra were referenced to the methyl resonance of TSP-*d*_4_ (*δ* 0.000) and bucketed into bins of 0.004 ppm in the range of *δ* 0.8-10.0 ppm. The regions of *δ* 4.700-5.116 were removed to eliminate the interference of imperfect water-saturation. All integrated bins were normalized to the peak area at *δ* 0.000 of each sample prior to statistical data analysis.

### 2.5. Lipidomic Profiling Based on UHPLC-MS Analysis

Three milligrams of heart powder were weighed and blended into 500 *μ*l water. The mixtures were homogenized via a TissueLyser-48 (Jingxin, Shanghai, China) at 60 Hz for 90 s. The homogenate was transferred into a 5 ml glass centrifuge tube and extracted with dichloromethane/methanol (2 : 1, *v*/*v*) twice based on the Cequier-Sánchez method [24]. Briefly, 490 *μ*l MeOH and 10 *μ*l MeOH containing 20 *μ*g/ml lysophosphatidylcholine (12 : 0) and 20 *μ*g/ml phosphatidylcholine (11 : 0/11 : 0) were added into the homogenate, and then 1 ml dichloromethane was added. Phase separation between aqueous and organic layers was performed by centrifugation at 3000 rpm for 15 min at room temperature. The organic phase at the bottom was collected into a new 5 ml glass centrifuge tube. The combined extractions were dried by vacuum, and the resultant powder was dissolved in 200 *μ*l isopropanol/methanol (1 : 1, *v*/*v*) and stored at -20°C.

Lipidomic analyses were conducted on an Ultimate®3000 ultra-high-performance liquid chromatography (UHPLC) system coupled to a Q Exactive Hybrid Quadrupole-Orbitrap MS system (Thermo Scientific, MA, USA). Separation was performed on a Hypersil GOLD C_18_ (100 × 2.1 mm, 1.9 *μ*m, Thermo Scientific, MA, USA), and 4 *μ*l of each sample was injected. The flow rate of the mobile phase and the temperature of the column oven were set to 0.35 ml/min and 45°C, respectively. The mobile phase comprised acetonitrile/water (3 : 2, 10 mM ammonium formate and 0.1% formic acid, phase A) and acetonitrile/isopropanol (1 : 9, 10 mM ammonium formate and 0.1% formic acid, phase B) following an optimal gradient: 0-14.5 min, 40%-100% B. All the samples were kept at 15°C during analyses. The MS raw data were acquired using the software Xcalibur (version 3.0, Thermo Scientific). LipidSearch (version 4.0, Thermo Scientific) was used for lipid identification and quantification according to the exact mass, retention time, and the pattern of precursor ions and MS^2^.

### 2.6. In Vivo Rat Myocardial Ischemia and Reperfusion

A total of 24 rats were randomly divided into three groups before ischemia-reperfusion (I/R) surgery. The rats in the treated group were administrated intragastrically with astragaloside IV at a dose of 40 mg/kg once daily for 7 days, while the rats in the sham and I/R groups received 10 ml/kg saline at the same time. After preadministration, rats were anesthetized with 5% chloral hydrate (6 ml/kg) and the chest was then opened. The proximal left anterior descending coronary artery (LADCA) was ligated with a 6/0 silk, which was released after 30 min allowing reperfusion to occur. The thorax was subsequently closed, and as soon as spontaneous respiration was sufficient, the rats were released and allowed to recover on an electric blanket. The animals in the sham group underwent the same procedure but without ligation on LADCA. A Vevo 2100 ultra-high-resolution small animal ultrasound imaging system (VisualSonics Vevo 2100, Canada) with a MS-250 ultrasound scanning transducer (model C5) was employed to acquire the parameters of the left ventricular function in real time, including the left ventricle ejection fraction percentage (EF%), aortic valve peak velocity (AV peak), +d*p*/d*t*_max_, −d*p*/d*t*_max_, and left ventricle fractional shortening percentage (FS%). At the end of the experiments, all data were analyzed off-line using the resident software in the ultrasound system. The hematoxylin-eosin (HE) stain was performed on the myocardium sections, and the levels of CK and LDH in the serum were determined by commercial kits.

### 2.7. Quantitative Real-Time PCR

Total RNA was extracted from myocardial tissue using a TRIzol reagent (Invitrogen, Carlsbad, CA), and DNase-treated RNA was reverse transcribed with the use of a Transcriptor First-Strand cDNA Synthesis Kit (Roche, Indianapolis, IN). Quantitative real-time PCR analysis was done in triplicate using the FastStart Universal SYBR Green Master (Roche). PCR primers were synthesized from Sangon Biotech Co. Ltd. (Shanghai, China) as follows: for superoxide dismutase (SOD), AGATGACTTGGGCAAAGGTG and CAATCCCAATCACACCACAA; for nuclear factor (erythroid-derived 2)-like 2 (Nrf2), TTCCTCTGCTGCCATTAGTCAGTC and GCTCTTCCATTTCCGAGTCACTG; for heme oxygenase-1 (HO-1), CACGCATATACCCGCTACCT and CCAGAGTGTTCATTCGAGCA; for catalases (CAT), ACATGGTCTGGGACTTCTGG and CCATTCGCATTAACCAGCTT; for Kelch-like ECH-associated protein 1 (Keap-1), AGCAGATCGGCTGCACTGAA and AGCTGGCAGTGTGACAGGTTG; for musculoaponeurotic fibrosarcoma (Maf), AAGGAGGAGGTGATCCGACT and TCGAGCAGTTTTCTCGGAAC; and for glyceraldehyde-3-phosphate dehydrogenase (GAPDH), ATGATTCTACCCACGGCAAG and CTGGAAGATGGTGATGGGTT. Among them, GAPDH was employed as a housekeeping gene. Relative mRNA expression levels were calculated by the 2^−*ΔΔ*Ct^ method.

### 2.8. Western Blot Analysis

After treatment and washing with cold PBS, myocardial tissue cut from the left ventricle surrounding (30 mg) was lysed in a radioimmunoprecipitation assay lysis buffer (Sangon Biotech Co. Ltd., Shanghai, China) to extract the whole protein. Tissue lysate was suspended in 400 *μ*l of cold buffer A (10 mM HEPES, pH 7.9, 10 mM KCl, 0.1 mM EDTA, 0.1 mM EGTA, 1 mM DTT, and 0.5 mM PMSF) and incubated for 15 min on ice followed by the addition of 50 *μ*l of 10% Nonidet P-40 and vortex for 10 s. After spinning for 30 s at 16,200 × g at 4°C, the pellet was saved and resuspended in 100 *μ*l of cold buffer C (20 mM HEPES, pH 7.91,400 mM KCl, 1 mM EDTA, 1 mM EGTA, 1 mM DTT, 1 mM PMSF, and 10 *μ*g/ml leupeptin/aprotinin) and kept on ice for 15 min. The mixture was spun again for 5 min, and the supernatant was collected as nuclear proteins and kept at -80°C until assay. The concentrations of the total protein in myocardial tissues and nuclei were determined by bicinchoninic acid (BCA) protein assay kit (Thermo Fisher Scientific, Waltham, USA). Protein samples were separated on SDS-PAGE gels and transferred to polyvinylidene difluoride membrane (Millipore, Bedford, MA). The membranes were probed with various primary antibodies overnight at 4°C. After incubation with secondary antibodies, the membranes were treated with ECL reagents. Protein levels were quantified with a VersaDoc MP 5000 multifunctional imaging analysis system (Bio-Rad Laboratories Inc., Hercules, CA, USA). Primary antibodies against protein kinase B (Akt, 4691, dilution ratio 1 : 500), phosphorylation Akt (p-Akt, 4060, dilution ratio 1 : 500), extracellular signal-regulated kinase 1/2 (ERK1/2, 4695, dilution ratio 1 : 500), phosphorylation ERK1/2 (p-ERK1/2, 4370, dilution ratio 1 : 500), HO-1 (82206, dilution ratio 1 : 1000), GAPDH (5174, dilution ratio 1 : 1000), and HRP-linked goat anti-rabbit/rat IgG secondary antibody (7074, dilution ratio 1 : 1000) were purchased from Cell Signaling Technology Inc. (MA, USA). The primary antibody against Nrf2 (sc-722) was purchased from Santa Cruz Biotechnology Inc. (CA, USA).

### 2.9. Statistical Analysis

Data were expressed as mean ± s.d., and *p* values were calculated using the two-tailed Student's *t*-test for pairwise comparisons and the one-way analysis of variance (ANOVA) for multiple comparisons. Comparisons between multiple-group means were performed using one-way analysis of variance (one-way ANOVA). Principal component analysis (PCA) and orthogonal partial least squares-discriminant analysis (OPLS-DA) were performed on SIMCA-P 14.1 software (Umetrics, Umea, Sweden).

## 3. Results

### 3.1. Astragaloside IV Prevents the Progress of Ischemia of Reperfusion Injury on the Heart

We first assessed the cardioprotective effect of astragaloside IV using the *ex vivo* (EV) Langendorff heart experiment. Twenty minutes of ischemia and 30 minutes of reperfusion resulted in myocardial injury in EV hearts, as evidenced by increasing infarct size (*p* < 0.01, Figure 1(b)), CK (*p* < 0.05), and LDH activities (*p* < 0.01, Figure 1(c)), along with decreasing cardiac function parameters, including HR, LVDP, and ±d*p*/d*t*_max_ (*p* < 0.01, Figure 1(d)). Astragaloside IV pretreatment significantly reduced infarct size (*p* < 0.01), CK (*p* < 0.01), and LDH (*p* < 0.05) releases in the EV hearts of the drug-treated group and also elevated systolic and diastolic function parameters (*p* < 0.01) compared with those in the corresponding I/R model group.

**Figure 2:**
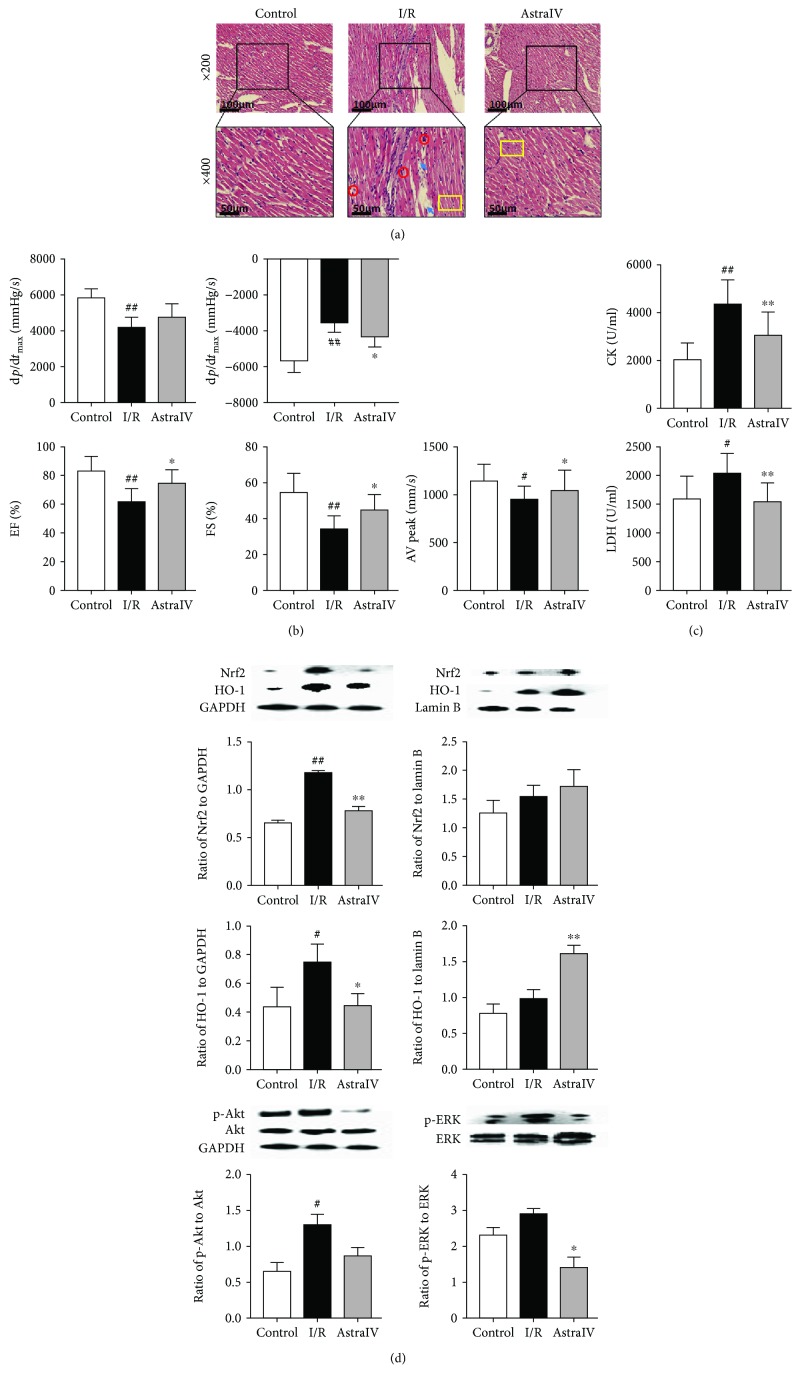


We next employed the *in vivo* (IV) rat model of myocardial ischemia and reperfusion to validate the efficiency of astragaloside IV. According to HE staining images (Figure 2(a)), myocardial edema, rupture of myocardial fibers, and infiltration of leukocytes were observed in the surrounding areas of infarction in the IV hearts of the I/R group, which were obviously prevented by using astragaloside IV in advance. Moreover, increase of EF%, FS%, AV peak, and ±d*p*/d*t*_max_ indicated that the cardiac function was significantly improved in the drug-treated group compared with the I/R group (*p* < 0.05, Figure 2(b)). Astragaloside IV also decreased the releases of CK and LDH in the serum (*p* < 0.01, Figure 2(c)). These results revealed that astragaloside IV protected both the EV and IV rat hearts against I/R injury.

### 3.2. Astragaloside IV Suppressed Cardiac Oxidation Stress by the Induction of Nrf2 Expression

Oxidative stress plays an important role in the ischemic cascade due to oxygen reperfusion injury following hypoxia. Since MDA is a lipid peroxidation marker of oxidative stress, we assessed its levels in EV rat hearts. As shown in Figure 1(e), astragaloside IV pretreatment significantly suppressed MDA levels (*p* < 0.05) and induced the activities of the antioxidant enzymes, SOD, and SDH (*p* < 0.01), in the myocardial tissues compared with the I/R group.

The transcription factor Nrf2 is considered to be one of the most important cellular defense mechanisms against oxidative stress with a particular role in the regulation of phase II detoxifying enzymes and antioxidant status. Its activation has been found to be primarily controlled by Keap1. Upstream kinases (e.g., Akt and ERK) activate Nrf2 through phosphorylation at specific sites favoring the release of Nrf2 from Keap1. Many endogenous enzymes (e.g., SOD, CAT, and HO-1) can be subsequently upregulated through the binding of Nrf2 to the antioxidant response element found in the promoters of these genes. We first quantified the expression of Keap1, Nrf2, HO-1, SOD, Maf, and CAT mRNA in EV hearts by qRT-PCR technology (Figure 1(f)). The results indicated that astragaloside IV pretreatment increased Keap1 (*p* < 0.05), SOD (*p* < 0.05), and CAT mRNA expressions and decreased Nrf2 (*p* < 0.05), HO-1 (*p* < 0.05), and Maf (*p* < 0.05) mRNA expressions compared with the I/R group.

We further measured the expression of Nrf2, HO-1, p-Akt, Akt, p-ERK, and ERK proteins in EV and IV hearts by the immunoblotting method (Figure 1(g) and Figure 2(d)). Compared to the sham-operated group, I/R injury stimulated Nrf2 and HO-1 protein expression in both cardiomyocytes and the nucleus. The ratios of p-Akt to Akt and p-ERK1/2 to ERK1/2 were also increased in the I/R group samples. Via preconditioning before I/R, astragaloside IV inhibited the total protein expression of Nrf2 and HO-1 in cardiomyocytes and decreased the ratios of p-Akt to Akt and p-ERK1/2 to ERK1/2, while promoting the protein levels of Nrf2 and HO-1 in the nucleus compared with the I/R group. It indicated that astragaloside IV probably activated the Nrf2/HO-1 pathway to exert antioxidant stress during the process of I/R injury.

### 3.3. Astragaloside IV-Adjusted I/R Injury Induced Metabolic Changes

The signal resonances in ^1^H NMR spectra of EV heart extracts from the control, I/R, and drug-treated groups were elucidated and assigned on the basis of chemical shift values and coupling constants and further confirmed by literature data and a range of 2D-NMR experiments (Table 1). Forty-six metabolites were identified in the extracts including amino acids, carbohydrates (glucose, mannose, and sucrose), TCA cycle intermediate (succinate), organic acid (lactate, 2-phenylpropionate), ketone bodies (3-hydroxybutyrate (3-HB)), purine and pyrimidine metabolites, nicotinurate, creatine, and choline metabolites.

**Figure 3:**
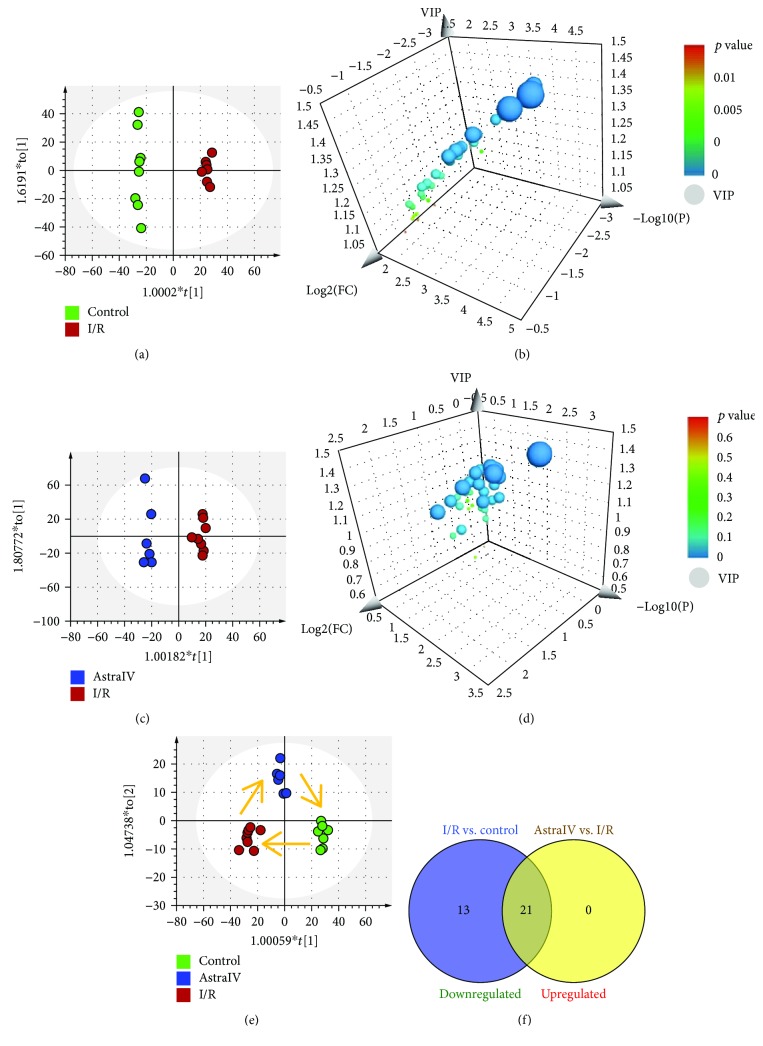


Multivariate analyses were conducted thoroughly for the NMR data obtained from myocardium tissue extracts. To identify the metabolites that significantly contributed to grouping, we performed OPLS-DA on normalized and unit-variance (UV) scaling data. The score plots from OPLS-DA showed significant metabolomic differences between control and I/R groups (*R*^2^*X* = 0.672, *R*^2^*Y* = 0.996, *Q*^2^ = 0.778; Figure 3(a)), as well as between I/R and drug-treated groups (*R*^2^*X* = 0.657, *R*^2^*Y* = 0.981, *Q*^2^ = 0.538; Figure 3(c)). With the screening conditions of VIP > 1 and *p* < 0.05, 34 metabolites were extracted and identified as potential biomarkers to distinguish the I/R group from the control group (Figure 3(b), Table 1), all of which were downregulated after I/R injury. Among them, 21 metabolites suggested significant intergroup differences between the I/R group and the drug-treated group and were upregulated with the pretreatment of astragaloside IV (Figure 3(d)–3(f), Table 1).

### 3.4. Astragaloside IV-reduced I/R Injury Induced Lipidomic Changes

**Figure 4:**
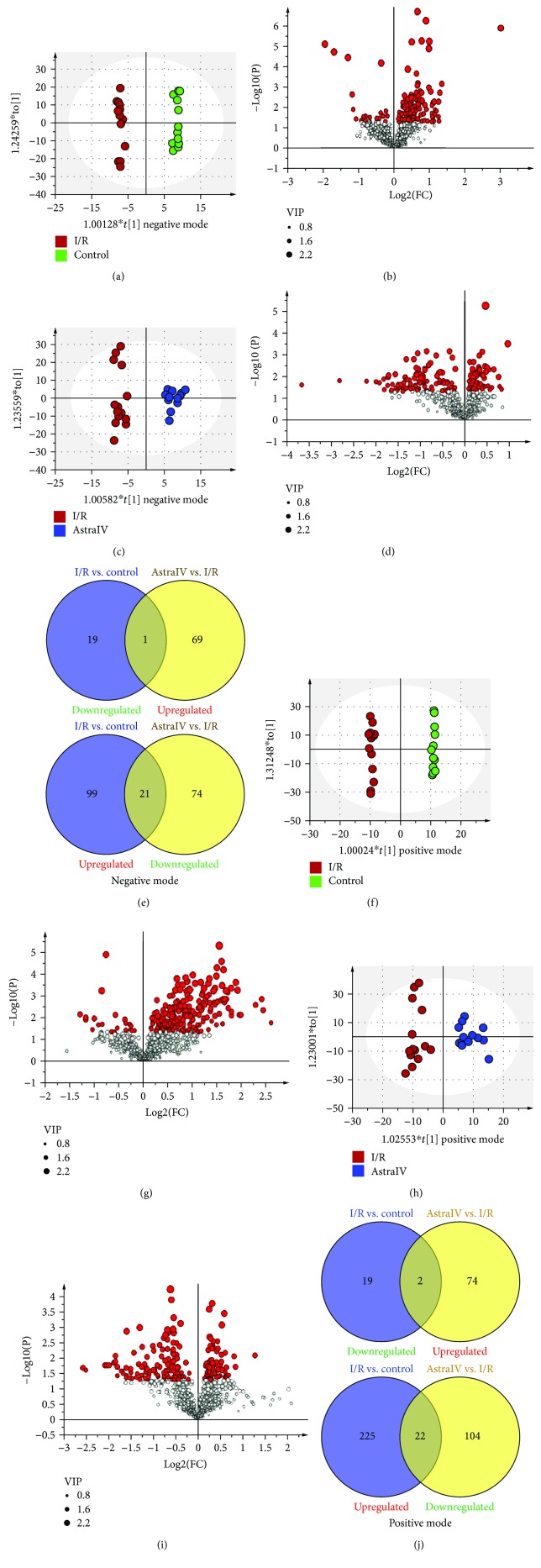


Lipid species from cardiolipin (CL), ceramide (Cer), fatty acids (FA), digalactosyl diacylglycerol (DGDG), lysophosphatidylcholine (LPC), lysophosphatidylethanolamine (LPE), lysophosphatidylglycerol (LPG), lysophosphatidylinositol (LPI), lysophosphatidylserine (LPS), monogalactosyl diacylglycerol (MGDG), (O-acyl)-hydroxy fatty acids (OAHFA), phosphatidic acid (PA), phosphatidylcholine (PC), phosphatidylethanolamine (PE), phosphatidylglycerol (PG), phosphatidylinositol (PI), phosphatidylserine (PS), sphingomyelin (SM), sphingosine (So), coenzyme (Co), diacylglycerol (DG), monoacylglycerol (MG), and triacylglycerol (TG) lipid categories were identified from the EV heart extracts, and the PCA score plots suggested that it was not necessary to remove any outlier of the data. Detailed lipidomic changes were obtained by constructing OPLS-DA models with the fitness and predictability expressed by the values of *R*^2^ and *Q*^2^. Differentiate ions in each pairwise comparison were selected by VIP value > 1 and *p* < 0.05. The score plots from OPLS-DA displayed a clear separation between the control and I/R groups in the negative (*R*^2^*X* = 0.500, *R*^2^*Y* = 0.994, and *Q*^2^ = 0.699; Figure 4(a)) and positive (*R*^2^*X* = 0.564, *R*^2^*Y* = 0.998, and *Q*^2^ = 0.649; Figure 4(f)) ion modes, respectively. Compared to the control group, 140 negative ions contributed to clustering and discrimination which were extracted, of which 20 ions were downregulated and 120 ions were upregulated in the I/R group (Figures 4(b) and 4(e)). Similarly, 268 positive ions were selected as differential variables, among which, 21 ions were downregulated and 247 ions were upregulated in the I/R group (Figures 4(g) and 4(j)). Removing duplicate lipids, a total of 343 lipid species were identified from these differential ions indicating the lipidomic changes induced by I/R injury in myocardium tissues.

To detect the modulation effect of astragaloside IV on lipid metabolism, OPLS-DA models were established between the drug-treated and I/R groups in the negative (*R*^2^*X* = 0.376, *R*^2^*Y* = 0.965, and *Q*^2^ = 0.577; Figure 4(c)) and positive (*R*^2^*X* = 0.419, *R*^2^*Y* = 0.916, and *Q*^2^ = 0.458; Figure 4(h)) ion modes, respectively. As a result, 165 negative and 202 positive ions were extracted by the decided thresholds (Figures 4(d) and 4(i)). Compared to the I/R group, 95 negative and 126 positive ions were downregulated, while 70 negative and 76 positive ions were upregulated under the pretreatment of astragaloside IV (Figures 4(e) and 4(j)). In particular, Venn diagrams revealed that 22 abnormal negative and 24 positive ions induced by I/R injury could be regulated back to normal levels with the pretreatment of astragaloside IV. Since 5 lipids were commonly detected in both positive and negative ion modes and 2 lipids were in the formation of a quasimolecular ion and other adduct molecular ion in the positive mode, a total of 39 differential lipids were improved with a regression trend by astragaloside IV (Table 2).

**Figure 5:**
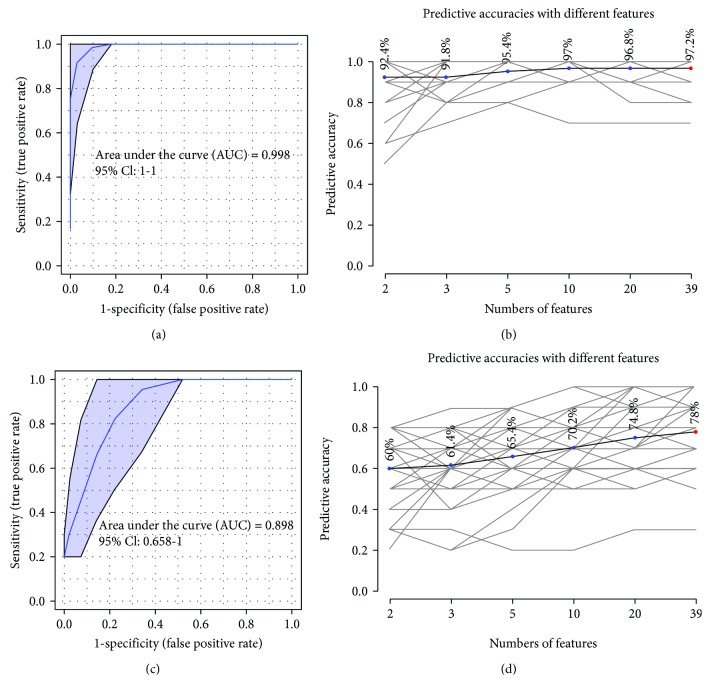


We next evaluated the performance of 39 lipid features using ROC curve analysis. The results showed that with the increasing numbers of top lipids in use, diagnosis of samples from the I/R or the control group was obviously improved. When all the 39 lipids were employed, the AUC value was nearly 1.0 with 95% confidence interval from 1 to 1 (Figure 5(a)) and a predictive value of 97.2% (Figure 5(b)). In the case of modeling between the astragaloside IV-treated group and the I/R group, the AUC value of 39 lipids was 0.898 with 95% confidence interval from 0.658 to 1 (Figure 5(c)) and a predictive value of 78% (Figure 5(d)). These values indicated identified that these 39 lipids were important features to astragaloside and can prevent and improve myocardial I/R injury.

## 4. Discussion

In the present work, we studied the efficacy and antioxidative mechanism of astragaloside IV against myocardial I/R injury. Astragaloside IV significantly increased the coronary blood flow and heart rate (HR), left ventricular development pressure (LVDP), and maximal ascending/descending rate (±d*p*/d*t*_max_) in the rats with I/R injury. At the same time, astragaloside IV reduced the damage degree of myocardial cells by decreasing the myocardial enzyme levels of CK and LDH in the coronary effluent and plasma.

Nrf2 is a member of the leucine zipper (bZIP) protein family, and it is also an important transcription factor that regulates the expression of antioxidant proteins that protect against oxidative damage triggered by injury and inflammation [25]. Under quiescent conditions, Nrf2 is anchored in the cytoplasm through binding to Keap1, and its effect is repressed by Keap1 [26]. When oxidative stress activates the dissociation of Nrf2 and Keap1, Nrf2 translocates into the nucleus and binds to the antioxidant response element (ARE) in the upstream promoter region of many antioxidative genes and then initiates their transcription [27]. In myocardial I/R rat models, we found that I/R injury caused increasing expression levels of Nrf2 protein in both tissues and the nucleus, while astragaloside IV preconditioning facilitated Nrf2 translocating into the nucleus and then promoted Nrf2 expression in the nucleus. RT-qPCR results showed that I/R injury increased the mRNA level of Nrf2, whereas drug pretreatment decreased the level. Similarly, compared with the control group, the ratios of p-Akt/Akt and p-ERK1/2/ERK1/2 were significantly increased in the I/R group, and they were decreased after astragaloside IV preconditioning. Nrf2 is bound to Keap1 in the cytoplasm; once activated, it dissociates and enters the nucleus to bind to Maf for stimulating the expression of downstream protein HO-1 [28]. As expected, Keap1 mRNA decreased in the I/R group compared with the control group, while it increased in the astragaloside IV pretreatment group (*p* < 0.01). Moreover, Maf and the downstream gene HO-1 in the I/R group were all significantly increased, while the SOD and CAT levels were lower than those in the control group. Notably, the expression of Nrf2 and HO-1 proteins in the nucleus all increased, and it showed a more obvious trend after astragaloside IV preconditioning, suggesting astragaloside IV might activate the antioxidative pathway of Keap1/Nrf2.

SOD and CAT are important enzymes in organisms with the function of scavenging oxygen free radicals. In the I/R group, the SOD level significantly decreased whereas it increased after drug intervention. SDH is an enzyme involved in cell oxidation and mitochondrial oxidative phosphorylation in the tricarboxylic acid (TCA) cycle [29]. The I/R group showed a significant reduction of SDH, which was improved in the drug-treated group, indicating that disturbance on TCA metabolism was prevented by astragaloside IV.

Succinate is an important metabolite of the TCA cycle. If a metabolic disorder occurs, it may affect the energy metabolism and related diseases of the body. It has been previously reported that succinate accumulates during ischemia, and the massive consumption of succinate during reperfusion can produce ROS [10]. Moreover, metabolomic analyses reveal that similar metabolic changes are observed in heart tissues of both *ex vivo* mouse Langendorff and *in vivo* ischemia and reperfusion experiments [10]. Since the *ex vivo* Langendorff heart is considered as a direct model for metabolomic analysis to observe ischemia and reperfusion injury, we thus employed *ex vivo* hearts to test the metabolic and lipidomic regulation effect of astragaloside IV. Using the ^1^H NMR-based metabolomic method here, we found that succinate was significantly reduced after reperfusion, and metabolites in the relevant metabolic pathways for succinate production were also reduced, which suggested that succinate consumption was likely to produce a large amount of ROS in the damaged myocardium after subsequent reperfusion. Via preconditioning with astragaloside IV, succinate was obviously increased and other related metabolites also recovered obviously, indicating that astragaloside IV probably prevented the production of excessive ROS. Therefore, succinate metabolism can be used as a new therapeutic target for myocardial reperfusion injury [30].

Overproduction of active oxidizing free radicals during I/R injury may cause lipid peroxidation, which is broadly defined as a process of inserting hydroperoxy groups into lipids. Polyunsaturated fatty acids (PUFA) present in glycerolipids, glycerophospholipids, and cholesterols are often the targets of peroxidation, of which the peroxyl groups are derived from an oxygen molecule or from hydrogen peroxide [31]. Lipid peroxidation has been hypothesized to be a major mechanism of free radical damage. It may alter intrinsic membrane properties due to the physicochemical changes of oxidized lipids or indirectly contribute to other deleterious effects of ischemia/reperfusion through enhancing phospholipid susceptibility for degradation by phospholipases [32, 33] and increasing membrane calcium permeability [34]. MDA is the metabolic intermediate of lipid peroxidation and has been recognized as an indicator for lipid peroxidation. We found that MDA increased significantly in the model group, indicating that I/R injury induced lipid peroxidation. Drug intervention decreased MDA obviously and revealed that astragaloside prevented lipid peroxidation by scavenging ROS.

Metabolomics has been an enabling technique for biomarker discovery and mechanism elucidation of coronary heart disease research [35]. Lipidomics, as a branch of metabolomics, has been used to investigate individual lipid species and their related metabolism pathway. A total of 39 lipids associated with I/R injury were remarkably improved by astragaloside IV, including LPC (15 : 0, 16 : 0, 16 : 1p, 17 : 0, 18 : 0, 18 : 1, 18 : 1p, 20 : 1, 20 : 3, 20 : 4, 22 : 4, and 22 : 6), PC (16 : 2/18 : 2, 17 : 0/18 : 2, 36 : 5, 38 : 4, and 41 : 4), LPE (17 : 0), PE (19 : 0e, 16 : 0/18 : 1, 18 : 0/20 : 5, and 39 : 5), LPG (16 : 0, 18 : 1), PG (17 : 0/18 : 1), LPI (18 : 1, 18 : 2, and 20 : 4), PS (37 : 3, 40 : 6), FA (24 : 5), CL (18 : 2/18 : 2/20 : 3/18 : 2), MG (22 : 6), DG (18 : 1/22 : 4, 22 : 6/22 : 6), and SM (d39 : 1, d22 : 0/18 : 1, d42 : 1, and d22 : 1/20 : 1). Although these lipids increased or decreased in different degrees induced by I/R injury, they were all regulated by astragaloside IV back to the normal levels of the control group. Among them, lysophospholipids (LPC, LPE, LPG, and LPI), as the products of plasmalogen reduction due to superoxide dismutase dysfunction, phospholipase activation, and oxidative stress [36–38], were found to be significantly downregulated in the drug-treated group, indicating astragaloside IV prevented oxidative stress damage and undue lipid peroxidation. The evidence was consistent with the results of biochemical factors. However, the causes and mechanisms of some other lipid changes have not yet been understood and still need further investigation.

**Figure 6:**
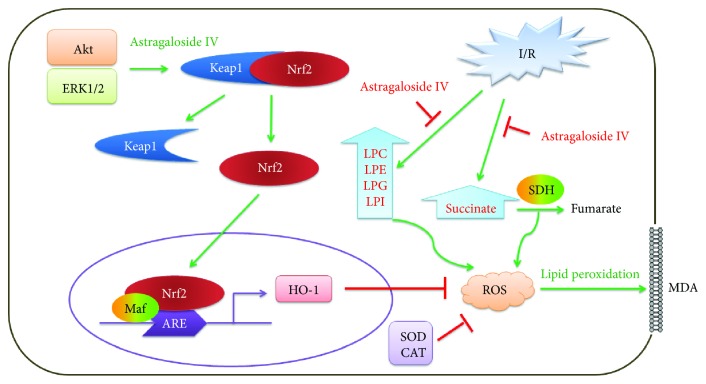


In summary, these results elucidate that astragaloside IV protects the myocardium from I/R injury through antioxidative stress, including preventing succinate accumulation to produce ROS, activating Nrf2 nuclear translocation to scavenge ROS, and avoiding lipid peroxidation caused by excessive ROS (Figure 6).

## Figures and Tables

**Table 1:** Metabolites identified from ^1^H NMR spectra and the differential ones contributed to group classification.

No.	Metabolites	Chemical shifts	Assignments	Model vs. control			Astragaloside IV vs. model		
				VIP value	*p* value	log2(FC)	VIP value	*p* value	log2(FC)
1	Isoleucine	0.94 (t), 1.02 (d), 1.26 (m), 1.47 (m), 1.98 (m), 3.67 (d)	*δ*-CH_3_, *γ*′-CH_3_, *γ*-CH_2_, *β*-CH, *α*-CH	1.2539	0.002	-0.9454	—	—	—
2	Leucine	0.95 (d), 0.96 (d), 1.71 (m), 3.74 (m)	*δ*, *δ*′-CH_3_, *β*-CH_2_, *γ*-CH, *α*-CH	1.0915	0.009	-1.1450	—	—	—
3	Valine	1.00 (d), 1.05 (d), 2.27 (m), 3.61 (d)	*γ*′-CH_3_, *γ*-CH_3_, *β*-CH, *α*-CH	1.2352	0.002	-1.1168	—	—	—
4	3-HB	1.22 (d), 2.31 (dd), 2.40 (dd), 4.15 (q)	CH_3_, CH_2_, CH	1.1132	0.007	-1.4905	—	—	—
5	Lactate	1.33 (d), 4.11 (q)	CH_3_, CH	1.3363	0.001	-1.9110	1.2059	0.047	1.0468
6	2-PP	1.43 (d), 3.64 (q)	CH_3_, CH	1.4707	0.001	-1.4671	1.2249	0.043	0.7255
7	Alanine	1.48 (d), 3.81 (m)	*β*-CH_3_, *α*-CH	1.0395	0.014	-0.9862	—	—	—
8	Acetate	1.92 (s)	CH_3_	—	—	—	—	—	—
9	Lysine	1.73 (m), 1.46 (m), 1.90 (m), 3.75 (m)	*δ*-CH_2_, *γ*-CH_2_, *β*-CH_2_, *α*-CH	1.1616	0.004	-0.9695	1.2591	0.036	0.4663
10	Glutamate	2.07 (m), 2.14 (m), 2.36 (m), 3.77 (m)	*β*-CH_2_, *γ*-CH_2_, *α*-CH	1.3332	0.001	-1.2331	1.2872	0.032	0.7509
11	Glutamine	2.15 (m), 2.46 (m), 3.80 (m)	*β*-CH_2_, *γ*-CH_2_, *α*-CH	1.3252	0.001	-1.5938	1.2292	0.042	0.9606
12	Succinate	2.42 (s)	CH_2_	1.317	0.001	-2.6171	1.4103	0.016	1.0785
13	Glutathione	2.56 (m), 2.15 (m), 3.76 (m)	*γ*-CH_2_, *β*-CH_2_, *α*-CH	1.2633	0.001	-1.2502	1.2127	0.046	0.6937
14	Aspartate	2.68 (dd), 2.81 (dd), 3.90 (m)	*β*-CH_2_, *α*-CH	1.4143	0.001	-2.8847	1.4946	0.009	1.5418
15	Asparagine	2.90 (dd), 4.00 (m)	*β*-CH_2_, *α*-CH	1.2156	0.002	-1.5568	—	—	—
16	TMA	2.92 (s)	CH_3_	—	—	—	—	—	—
17	Creatine	3.04 (s), 3.93 (s)	CH_3_, CH_2_	1.0443	0.013	-1.3208	1.3512	0.022	0.4632
18	Choline	3.21 (s)	-N(CH_3_)_3_	1.2029	0.003	-1.0859	—	—	—
19	GPC	3.22 (s)	-N(CH_3_)_3_	1.0832	0.009	-1.1119	—	—	—
20	Carnitine	3.23 (s)	-N(CH_3_)_3_	1.0921	0.009	-1.2129	1.3242	0.026	0.5116
21	Taurine	3.26 (t), 3.43 (t)	N-CH_2_, S-CH_2_	1.2997	0.001	-1.4200	1.3163	0.027	1.0468
22	Methanol	3.36 (s)	CH_3_	—	—		—	—	—
23	Glycerol	3.56 (dd), 3.65 (dd), 3.79 (m)	CH_2_, CH	1.1959	0.003	-1.3804	1.7648	0.001	0.8321
24	Glycine	3.56 (s)	−CH_2_−NH_2_	1.2479	0.002	-1.0945	—	—	—
25	Inosine	3.84 (dd), 3.91 (dd), 4.28 (m), 4.44 (dd), 6.11 (d), 8.23 (s), 8.35 (s)	17-CH_2_, 5-CH, 4-CH, 2-CH, 12-CH, 7-CH	1.2668	0.001	-1.8160	1.3022	0.029	0.7268
26	Serine	3.96 (m); 3.84 (m)	*β*-CH_2_, *α*-CH	1.1686	0.004	-1.2163	1.4665	0.011	0.6898
27	Threonate	4.01 (d), 3.97 (m), 3.63 (dd)	2-CH, 3-CH, 4-CH_2_	1.3418	0.001	-1.3562	1.5293	0.007	0.9188
28	Threonine	1.32 (d), 4.22 (m), 3.58 (m)	*γ*-CH_3_, *β*-CH, *α*-CH	1.4887	0.001	-1.7117	1.5064	0.008	0.9664
29	Glucose	5.24 (d), 4.65 (d)	(*α*) 1-CH, (*β*) 1-CH	1.332	0.001	-1.4715	1.4316	0.014	1.0181
30	Mannose	5.19 (d), 4.89 (d)	(*α*) 1-CH, (*β*) 1-CH	1.2556	0.002	-2.3335	1.2752	0.034	1.2687
31	Sucrose	5.40 (d)	(Glucose) 1-CH	1.1204	0.007	-3.4659	—	—	—
32	Cytidine	6.05 (d), 7.83 (d)	5-CH, 6-CH	1.2756	0.001	-1.5560	1.4658	0.011	1.9998
33	Uracil	5.80 (d), 7.53 (d)	5-CH, 6-CH	—	—	—	—	—	—
34	Guanosine	5.90 (d), 8.00 (s)	2-CH, 11-CH	1.1306	0.006	-3.1140	1.3569	0.022	1.5474
35	Fumarate	6.52 (s)	CH	—	—	—	—	—	—
36	Tyrosine	6.89 (m), 7.19 (m)	2-CH, 6-CH, 3-CH, 5-CH	1.1655	0.004	-0.9610	—	—	—
37	Histidine	7.11 (s), 7.89 (s)	5-CH, 2-CH	—	—	—	—	—	—
38	Phenylalanine	7.33 (m), 7.38 (m), 7.42 (m)	2-CH, 6-CH, 4-CH, 3-CH, 5-CH	1.2019	0.003	-0.7999	—	—	—
39	Nicotinurate	7.60 (m), 8.24 (m), 8.72 (m), 8.94 (m)	5-CH, 4-CH, 6-CH, 2-CH	1.3414	0.001	-1.1851	—	—	—
40	Uridine	5.89 (d), 7.87 (d)	5-CH, 6-CH	1.4161	0.001	-2.1078	1.6107	0.004	1.2031
41	Xanthine	7.96 (s)	2-CH	—	—	—	—	—	—
42	Oxypurinol	8.19 (s)	9-CH	—	—	—	—	—	—
43	AMP	6.13 (d), 8.22 (s), 8.58 (s)	2-CH, 12-CH, 7-CH	—	—	—	—	—	—
44	ADP	6.13 (d), 8.26 (s), 8.52 (s)	2-CH, 12-CH, 7-CH	—	—	—	—	—	—
45	ATP	6.13 (d), 8.26 (s), 8.51 (s)	2-CH, 12-CH, 7-CH	—	—	—	—	—	—
46	Formate	8.46 (s)	CHO	—	—	—	—	—	—

VIP: variable important for the projection; FC: fold change; 3-HB: 3-hydroxybutyrate; 2-PP: 2-phenylpropionate; TMA: trimethylamine; GPC: glycerophosphocholine; AMP: adenosine monophosphate; ADP: adenosine diphosphate; ATP: adenosine triphosphate.

**Table 2:** Differential lipid species contributed to group classification.

No.	Lipid	Lipid ion	Model vs. control			Astragaloside IV vs. model		
			VIP value	*p* value	log2(FC)	VIP value	*p* value	log2(FC)
1	DG (18 : 1/22 : 4)	DG (18 : 1/22 : 4) +NH4	2.2076	0.001	1.6410	1.4538	0.040	-0.6451
2	CL (18 : 2/18 : 2/20 : 3/18 : 2)	CL (18 : 2/18 : 2/20 : 3/18 : 2)-H	2.5225	0.001	0.4739	1.4087	0.035	-0.2150
3	DG (22 : 6/22 : 6)	DG (22 : 6/22 : 6) +NH4	1.2593	0.008	-1.2863	1.4958	0.008	1.2987
4	FA (24 : 5)	FA (24 : 5)-H	1.8095	0.004	0.6340	1.5863	0.016	-0.4651
5	LPC (15 : 0)	LPC (15 : 0) +H	1.4051	0.023	0.5568	1.7333	0.013	-0.5419
6	LPC (16 : 0)	LPC (16 : 0) +H	1.4575	0.017	0.4393	1.8399	0.008	-0.4683
		LPC (16 : 0) +HCOO	1.8306	0.003	0.4709	1.4347	0.031	-0.2877
7	LPC (16 : 1p)	LPC (16 : 1p) +H	1.6036	0.008	0.5349	1.8856	0.006	-0.5417
8	LPC (17 : 0)	LPC (17 : 0) +H	1.8525	0.007	-0.5395	1.4751	0.016	0.4943
		LPC (17 : 0) +HCOO	2.0689	0.001	0.6604	1.5609	0.018	-0.3770
9	LPC (18 : 0)	LPC (18 : 0) +H	1.5194	0.013	0.4179	1.5409	0.029	-0.3576
10	LPC (18 : 1)	LPC (18 : 1) +H	1.3712	0.026	0.7205	1.8124	0.009	-0.7992
		LPC (18 : 1) +HCOO	2.0410	0.001	0.6449	1.5586	0.018	-0.4051
11	LPC (18 : 1p)	LPC(18 : 1p) +H	1.6013	0.008	0.6067	1.6208	0.021	-0.4767
12	LPC (20 : 1)	LPC (20 : 1) +HCOO	1.5674	0.014	0.4916	1.7422	0.007	-0.5742
13	LPC (20 : 3)	LPC (20 : 3) +H	1.5281	0.012	0.7497	1.7114	0.014	-0.5899
14	LPC (20 : 4)	LPC (20 : 4) +H	1.7039	0.004	0.6532	1.6392	0.019	-0.4448
		LPC (20 : 4) +HCOO	1.8538	0.003	0.6563	1.5171	0.022	-0.3879
15	LPC (22 : 4)	LPC (22 : 4) +H	1.7633	0.003	0.7929	2.0677	0.002	-0.7530
16	LPC (22 : 6)	LPC (22 : 6) +H	1.7244	0.004	0.6828	1.4231	0.045	-0.3728
		LPC (22 : 6) +HCOO	1.9491	0.002	0.7185	1.3837	0.038	-0.3549
17	LPE (17 : 0)	LPE (17 : 0) +H	1.3783	0.026	0.3498	1.7758	0.010	-0.5035
18	LPG (16 : 0)	LPG (16 : 0)-H	1.5975	0.012	1.1349	1.7168	0.008	-1.0794
19	LPG (18 : 1)	LPG (18 : 1)-H	1.3321	0.041	0.8443	1.7138	0.008	-0.9971
20	LPI (18 : 1)	LPI (18 : 1)-H	1.3081	0.045	1.0946	1.3901	0.037	-1.0277
21	LPI (18 : 2)	LPI (18 : 2)-H	1.4379	0.026	1.2306	1.5986	0.015	-1.2328
22	LPI (20 : 4)	LPI (20 : 4)-H	1.4179	0.029	1.0758	1.3296	0.047	-0.8204
23	MG (22 : 6)	MG (22 : 6) +H	1.3515	0.029	0.4772	1.6409	0.019	-0.6263
24	PC (16 : 2/18 : 2)	PC (16 : 2/18 : 2) +HCOO	1.0640	0.042	-1.0172	1.0495	0.043	0.7557
25	PC (17 : 0/18 : 2)	PC (17 : 0/18 : 2) +HCOO	1.4689	0.023	0.7415	2.0816	0.001	-1.0504
26	PC (36 : 5)	PC (36 : 5) +H	2.0056	0.001	1.8005	2.1950	0.001	-1.2854
27	PC (38 : 4)	PC (38 : 4) +H	1.3104	0.035	0.4454	1.7385	0.012	-0.5253
28	PC (41 : 4)	PC (41 : 4) +H	2.2853	0.001	1.6044	1.9778	0.004	-0.8453
29	PE (16 : 0/18 : 1)	PE (16 : 0/18 : 1)-H	1.7538	0.005	0.4561	1.7541	0.007	-0.4875
30	PE (18 : 0/20 : 5)	PE (18 : 0/20 : 5)-H	1.2958	0.048	1.0505	1.6508	0.012	-1.3285
31	PE (39 : 5)	PE (39 : 5) +H	1.9500	0.001	1.7287	1.5870	0.024	-0.9653
32	PE (19 : 0e)	PE (19 : 0e) +H	1.4636	0.017	0.4501	1.7815	0.010	-0.4539
33	PG (17 : 0/18 : 1)	PG (17 : 0/18 : 1)-H	1.3876	0.033	0.1410	2.0119	0.001	-0.2670
34	PS (37 : 3)	PS (37 : 3)-H	2.6293	0.001	3.0100	1.3271	0.048	-0.4890
35	PS (40 : 6)	PS (40 : 6) +H	1.3239	0.008	-0.5606	1.1997	0.008	0.5361
36	SM (d22 : 1/20 : 1)	SM (d22 : 1/20 : 1) +HCOO	2.6812	0.001	0.8807	1.5137	0.022	-0.2800
37	SM (d39 : 1)	SM (d39 : 1) +HCOO	1.8395	0.003	0.4619	1.4322	0.031	-0.3028
38	SM (d42 : 1)	SM (d42 : 1) +H	1.6231	0.007	0.5924	1.3941	0.050	-0.4197
39	SM (d22 : 0/18 : 1)	SM (d22 : 0/18 : 1) +HCOO	2.7354	0.001	0.6439	2.1077	0.001	-0.3292

VIP: variable important for the projection; FC: fold change; DG: diacylglycerol; MG: monoacylglycerol; CL: cardiolipin; FA: fatty acid; LPC: lysophosphatidylcholine; PC: phosphatidylcholine; LPE: lysophosphatidylethanolamine; PE: phosphatidylethanolamine; LPI: lysophosphatidylinositol; PS: phosphatidylserine; PG: phosphatidylglycerol; LPG: lysophosphatidylglycerol; SM: sphingomyelin.

## Data Availability

The data used to support the findings of this study are included within the article.
